# Sleep problems in prostate cancer patients: a comparison of several questionnaires measuring sleep quality

**DOI:** 10.3389/fpsyg.2026.1729459

**Published:** 2026-02-11

**Authors:** Andreas Hinz, Thomas Schulte, Michael Friedrich, Jochen Ernst, Katja Petrowski, Anja Mehnert-Theuerkauf

**Affiliations:** 1 Department of Medical Psychology and Medical Sociology, Comprehensive Cancer Center Central Germany, University Medical Center Leipzig, Leipzig, Germany; 2 Rehabilitation Clinic Bad Oexen, Bad Oeynhausen, Germany; 3 Department of Medical Psychology and Medical Sociology, University Medical Center of the Johannes Gutenberg University Mainz, Mainz, Germany

**Keywords:** insomnia, prostate cancer, quality of life, questionnaire, sleep

## Abstract

**Objective:**

Prostate cancer (PCa) patients often suffer from sleep problems. The aims of this study were to compare several questionnaires for measuring sleep problems, to compare the sleep problems of PCa patients with those of the general population, to calculate associations between sleep problems and other variables, and to analyze the factor structure of sleep items.

**Methods:**

A sample of 309 PCa patients treated in a German rehabilitation hospital were examined. Their sleep problems were assessed with the Pittsburgh Sleep Quality Index (PSQI), the Insomnia Sleep Scale (ISI), the Jenkins Sleep Scale (JSS), and two single-item measures. In addition, questionnaires on quality of life, anxiety, depression, and fear of cancer recurrence were used.

**Results:**

Sleep quality in the PCa group was markedly worse than that of the general population, with effect sizes between 0.69 and 0.97. The correlations between the sleep scales ranged from 0.64 to 0.84, which indicates a certain but not complete interchangeability. Sleep problems were associated with anxiety, depression, and fear of cancer recurrence. A principal component analysis yielded three factors underlying the items of the sleep instruments.

**Conclusion:**

The study confirmed sleep problems in PCa patients. Results obtained by using one specific questionnaire can only be partly generalized to other instruments.

## Introduction

Worldwide, prostate cancer (PCa) is the most frequently diagnosed cancer in men. It accounts for about 15% of all male cancers (Sung et al., 2021; Ferlay et al., 2021). Regarding cancer mortality, PCa has the second highest mortality after lung cancer in men (Bray et al., 2024). The Lancet Consortium predicted that the annual frequency of new PCa cases will increase from 1.4 million in 2020 to 2.9 million by 2040 (James et al., 2024).

Sleep disturbances are a significant concern among PCa patients (Savard et al., 2013). The prevalence of sleep problems in this group is notably higher than in the general population, with estimates ranging from 30% to 75% for newly diagnosed or recently treated patients (Mondal et al., 2022).

Sleep problems in PCa patients are associated with reduced quality of life (QoL), distress, anxiety and depression, pain, fatigue (Mangar et al., 2023; Sparasci et al., 2022), side effects such as urinary symptoms and intestinal symptoms (Maguire et al., 2019), frailty (Liang et al., 2025), and even increased mortality (Sparasci et al., 2022; Tan et al., 2018; Jan et al., 2016). In a study comparing different dimensions of QoL with regard to the difference between cancer patients and the general population, sleep problems showed the second largest detrimental effect after fatigue (Hinz et al., 2018).

Various factors contribute to sleep problems, including the effects of cancer treatment, psychological stressors, and physiological changes associated with the disease. Androgen deprivation therapy (ADT), radiotherapy, and surgical interventions play a role in sleep issues, likely through mechanisms involving inflammatory cytokines and the psychological burden of the cancer diagnosis (Sparasci et al., 2022; Mangar et al., 2023). Specific studies have shown that PCa patients undergoing radiation therapy experience significant sleep disruptions, particularly regarding sleep latency and overall sleep quality (Thomas et al., 2010; Garrett et al., 2011). A systematic review summarized the impact of active treatment methods on sleep disruptions in PCa patients and their impact on QoL (Sparasci et al., 2022).

Sleep disorders such as obstructive sleep apnea have also been linked to a significantly higher risk of developing PCa, underscoring the complex interplay between sleep health and cancer outcomes (Wiggins et al., 2020; Chung and Lin, 2019). Biologically, disruptions in sleep patterns can lead to alterations in melatonin production, which are linked to cancer development and progression. Sleep problems may affect PCa progression due to potential circadian rhythm disruptions influencing tumor biology (Sigurdardottir et al., 2013). The relationship between sleep problems and increased risk for high-grade PCa was shown in a gene correlation analysis, reinforcing the connection between sleep disorders and cancer pathology (Liu et al., 2025).

Despite their high prevalence, sleep problems are often not recognized in the treatment process because health care providers do not ask about sleep issues and patients do not mention them on their own initiative (Mercadante et al., 2017). This means that opportunities to alleviate sleep problems remain unexploited. The use of short sleep screening instruments can help to remedy this situation.

Multiple instruments have been developed for effectively measuring sleep problems, e.g., the Pittsburgh Sleep Quality Index (PSQI) (Buysse et al., 1989), the Insomnia Severity scale (ISI) (Morin et al., 2011), and the Jenkins Sleep Scale (JSS) (Jenkins et al., 1988). Moreover, some questionnaires that focus on related topics, such as QoL, mental health or depression, contain one item that is related to sleep quality, e.g., the EORTC QLQ-C30 (Aaronson et al., 1993) and the Patient Health Questionnaire-9 (PHQ-9) (Kroenke et al., 2001).

The questionnaires on sleep problems differ with respect to several features: the specific content and the number of items, the time horizon, and the response format. Most studies of sleep problems in cancer patients use only one questionnaire for assessing sleep quality. In these cases, it remains unclear to what degree the results obtained with the specific questionnaire depend on the chosen instrument. Only few examinations used more than one instrument and compared the results obtained with these instruments. For example, a study with the ISI and the sleep item of the PHQ-9 reported a correlation of *r* = 0.72 between these two assessment instruments in a sample of cancer patients (Schulte et al., 2021); a further study correlated the results of the JSS and the sleep item of the EORTC QLQ-C30 (*r* = 0.73) (Hofmeister et al., 2020). This means that, although the different sleep quality questionnaires measure the same thing to a certain extent, they are not interchangeable. For this reason, three different questionnaires for recording sleep quality were used in parallel in our study. The correlations between the questionnaires were intended to provide information on the extent to which the different instruments actually measure the same variable, and thus the extent to which the results obtained with a specific instrument can be generalized.

The term sleep quality encompasses several aspects of sleep: the subjectively experienced sleep quality, sleep duration, sleep disturbances, time between going to bed and falling asleep, number of awakenings in the night, and sleep efficiency. The PSQI explicitly comprises seven separate dimensions, one of them being subjective overall sleep quality. These dimensions are more or less correlated. For better understanding the processes underlying the general judgments on sleep problems, it is useful to test whether there are fundamental factors that build sleep quality. A study with breast cancer patients included a factor analysis with the seven components of the PSQI and arrived at a two-factor solution (Fontes et al., 2017), while a study with a large general population sample reported that a three-factor solution fitted the data best (Jia et al., 2019). Based on the items of the ISI, several studies with very diverse populations investigated the factor structure and reported factor numbers from 1 to 3 (Yusufov et al., 2019). The factor structure of a larger set of sleep-related items, based on a sample of PCa patients, has not been investigated yet.

The aims of this study were (a) to investigate the prevalence of sleep problems in PCa patients in comparison with sleep problems in men from the general population, (b) to analyze the associations between sleep problems and further variables, such as QoL, anxiety, depression, fear of cancer recurrence, and health anxiety, (c) to compare three established questionnaires and two one-item scales for measuring sleep problems in the context of the above noted research questions, and (d) to analyze the factor structure of the items included in the sleep instruments.

## Methods

### Sample of cancer patients

In Germany, cancer patients are offered the opportunity to participate in rehabilitation programs to help restore their physical, mental and social functioning. Between July 2022 and June 2023, study participants were consecutively recruited in a German oncological rehabilitation hospital. The inclusion criteria for this study were: a confirmed cancer diagnosis, age 18 years and above, absence of severe cognitive impairment, and sufficient command of the German language. A total of 2,250 patients with any cancer diagnosis were eligible and therefore asked to take part in the study, and 1,733 (77 %) of them agreed to participate. Written informed consent was obtained from all participants. The study was approved by the Ethics Committee of the Medical Faculty of the University of Leipzig, approval number: 513/21-ek. From this sample of cancer patients, we only considered PCa patients in the further analyses.

### Instruments

The following instruments were used in the study:

#### PSQI

The Pittsburgh Sleep Quality Index (PSQI) (Buysse et al., 1989) comprises 19 items that can be assigned to the following seven dimensions: subjective sleep quality, sleep latency, sleep duration, sleep efficiency, sleep disturbances, use of sleep medication, and daytime dysfunction, with different numbers of items per dimension. For each dimension, the items are summarized and transformed to a value between 0 and 3. For the total score of overall sleep quality, these seven individual scores are added together, resulting in a sum score range from 0 to 21, whereby high scores indicate high levels of sleep problems. Sum scores ≥ 6 are generally considered as indicating poor sleep (Buysse et al., 1989). Normative scores of the PSQI are available (Hinz et al., 2017a).

#### ISI

The development of the Insomnia Severity Index (ISI) (Morin et al., 2011; Dieck et al., 2018) was based on the diagnostic criteria for insomnia outlined in the Diagnostic and Statistical Manual of Mental Disorders (DSM-IV) and the International Classification of Sleep Disorders (ICSD). The instrument consists of seven items as indicators of insomnia: sleep onset, sleep maintenance, early morning awakening, satisfaction level with current sleep pattern, interference with daily living, noticeability of impairment due to the sleep difficulty, and level of distress caused by the sleep problem. For each item, there are five response options, coded as 0–4, resulting in a sum score ranging from 0 to 28. The ISI sum scores can be assigned to categories as follows: no significant insomnia (0–7), subthreshold insomnia (8–14), moderate insomnia (15–21), and severe insomnia (22–28) (Morin et al., 2011).

#### JSS

The Jenkins Sleep Scale (JSS) (Jenkins et al., 1988) is an instrument for measuring sleep problems with the following four items: trouble falling asleep, waking up several times per night, trouble staying asleep, and waking up tired. For each item, there are six response options, coded from 0 to 5, which results in a sum score range from 0 to 20. Though there is no generally accepted cutoff for poor sleep quality, proposals for cutoffs have been made, e.g., ≥ 12 for sleep problems (Monterrosa-Castro et al., 2016), or 0–9 (none/some), 10–14 (moderate), 15–20 (severe sleep problems) (Gianfagna et al., 2016). Normative values of the JSS are available (Tibubos et al., 2020).

#### EORTC QLQ-C30

The EORTC QLQ-C30 (Aaronson et al., 1993) is an instrument for measuring QoL in cancer patients. It consists of 30 items that are assigned to five functioning scales, a global health status/QoL scale, three symptom scales, and six single-item scales. Each scale of the EORTC QLQ-C30 is transformed to the range from 0 to 100, with higher functioning scores and lower symptom scores representing better QoL. One of these single-items scales refers to sleep problems with the question “Have you had trouble sleeping?”. Normative values of the EORTC QLQ-C30 are available (Nolte et al., 2019; Hinz et al., 2014).

In addition to the sleep scales and the QoL questionnaire, the study included the Generalized Anxiety Disorder Screener (GAD-7) (Löwe et al., 2008) for measuring anxiety with seven items and the Patient Health Questionnaire-9 (PHQ-9) (Kroenke et al., 2001) for measuring depression with nine items. One of the items of the PHQ-9 refers to sleep problems. Moreover, the study included the 12-item Fear of Progression Questionnaire (FoP-Q-SF) (Mehnert et al., 2009, 2006), the 4-item Concerns About Recurrence Questionnaire (CARQ-4) (Thewes et al., 2015), and the Whiteley Index (WI-7) measuring health anxiety with seven items (Carstensen et al., 2020).

### Statistical analysis

The association between the sleep scales as well as the associations between the sleep scales and the other scales were expressed in terms of Pearson correlations. The reliability (internal consistency) of the sleep scales was determined with Cronbach's α coefficient. Effect sizes *d* were calculated to indicate group mean differences. These coefficients relate the difference between the group means to the pooled standard deviation. If at least 75 % of the items of a scale were valid, the missing values were replaced by the mean of the valid values; otherwise, the patient was excluded from the analysis.

To compare the patients' scores with those of the general population, we used normative data taken from large representative general population studies for the EORTC QLQ-C30 (Hinz et al., 2014), the GAD-7 (Löwe et al., 2008) and the PHQ-9 (Kocalevent et al., 2013). We used the following strategy: from the publication of the normative scores of the EORTC QLQ-C30, based on six European studies, we used the men's mean scores and standard deviations of the age decades as reported in the normative tables. The age decades were weighted according to the frequencies of the age decades in our PCa sample. For example, the percentage of patients in the age group 60–69 years was 49.8 % in this study. These percentages were taken as the weighing factors for calculating the general population mean value of the scales of the EORTC QLQ-C30.

Since we had no hypothesis on the factor structure of the sleep items, we conducted an exploratory factor analysis (EFA). All items of the ISI and the JSS, the subscales of the PSQI, and the two single sleep items of the EORTC QLQ-C30 and the PHQ-9 were included. Factors were extracted using principal component analysis (PCA). To account for inter-correlated factors, we chose varimax rotation. With the Kaiser-Meyer-Olkin (KMO) test, we verified whether the common variance of the items was at least as large as their specific variance. The test provides a measure of sampling adequacy, of which values below 0.5 are considered unacceptable. The number of factors was determined using the Kaiser-Guttman criterion (eigenvalues > 1). All calculations were performed with SPSS, version 27.

## Results

### Sample characteristics

A total of 2,250 cancer patients of all diagnoses were eligible to take part in the wider study, and 1,733 (77 %) of these agreed to participate and to complete the questionnaires. Of these, there were 309 men with PCa. In this study, we restricted the analyses to these patients. The number of missing values for the items ranged from 0 (0.0 %) to 6 (1.6 %). The reason for this low number of missing values is that the study nurse checked each completed questionnaire and, if possible, asked the patients to complete any missing information.

The mean age of the 309 PCa patients was 66.4 years (SD = 7.0 years) with a range from 48 years to 83 years. Further characteristics are given in Table 1. Most of the patients were retired and received surgery. The frequencies of chemotherapy, radio therapy, hormone therapy, and antibody therapy were low.

**Table 1:** Sociodemographic and clinical characteristics of the sample (*n* = 309).

**Characteristics**	***n***	**%**
**Age group**		
18–59 years	49	15.9
60–69 years	154	49.8
≥70 years	106	34.3
**Education^a^**		
Elementary school (8–9 years)	111	36.2
Junior high school (10 years)	76	24.8
High school/university (≥11 years)	118	38.4
No formal qualification	2	0.7
**Employment status^a^**		
Employed	106	34.6
Unemployed	4	1.3
Retired	195	63.7
Other	1	0.3
**Time since diagnosis**		
≤ 12 months	233	75.4
> 12 months	76	24.6
**Treatment**		
**Surgery^a^**		
No	16	5.2
Yes	293	94.8
**Chemotherapy^a^**		
No	299	97.1
Yes	9	2.9
**Radio therapy^a^**		
No	260	84.1
Yes	49	15.9
**Hormone therapy^a^**		
No	258	84.3
Yes	48	15.7
**Antibody therapy^a^**		
No	303	98.7
Yes	4	1.3

^a^Missing data not reported.

### Sleep scale mean scores and comparisons with the general population

Table 2 presents the mean scores of the five sleep-related scales and several other scales for the PCa patients. For those scales for which norm values were available, the corresponding values for the general male population are also given. The effect size refers to the group difference in mean value relative to the standard deviation. Because there are no normative values for the ISI, FoP-Q-SF, CARQ-4, and WI, group comparisons cannot be performed for these instruments. The highest effect sizes were obtained for sleep problems measured with the JSS (*d* = 0.97) and the sleep scale of the EORTC QLQ-C30 (*d* = 0.91).

**Table 2:** Comparison between PCa patients and general population samples.

	**PCa patients**		**General population**		***d***
**Scales**	**M**	**(SD)**	**M**	**(SD)**	
**Sleep scales**					
PSQI	6.82	(3.92)	4.39	(3.09)	0.69
ISI	9.56	(6.16)	–	–	–
JSS	9.36	(5.17)	4.54	(4.75)	0.97
EORTC QLQ-C30-sleep	41.53	(34.15)	15.02	(23.90)	0.91
PHQ-9-sleep	1.20	(1.01)	0.61	(0.47)	0.80
EORTC QLQ-C30 (quality of life)					
Global health/QoL	65.37	(18.42)	68.90	(19.61)	−0.19
Sum score	75.77	(15.14)	89.06	(14.90)	−0.88
PHQ-9 (Depression)	4.54	(4.10)	3.16	(3.67)	0.36
GAD-7 (Anxiety)	3.96	(3.90)	2.73	(3.09)	0.35
FoP-Q-SF (Fear of progression)	23.29	(7.94)	–	–	–
CARQ-4 (Fear of recurrence)	11.01	(9.42)	–	–	–
Whiteley Index (Health anxiety)	75.77	(15.14)	–	–	–

M, mean; SD, standard deviation; *d*, effect size of the group difference.

After applying the usual cutoff values to categorize sleep quality, the following frequencies were obtained for the PCa patients: PSQI (scores above 5): 166 patients (53.7 %) poor sleep; JSS (cutoff of 12): 114 patients (36.9 %) poor sleep; and ISI: 134 patients (43.4 %) no significant insomnia, 110 patients (35.6 %) subthreshold insomnia; 49 (15.9 %) moderate insomnia, and 16 (5.2 %) severe insomnia.

### Correlations between the sleep scales and other scales

The correlations between the sleep scales are given at the top of Table 3. The strongest association was found for the relationship between ISI and JSS (*r* = 0.84), while the weakest correlation was found for the two single-item scales C30-sleep and PHQ-9-sleep (*r* = 0.64).

**Table 3:** Correlations between the sleep scales and correlations between sleep scales and other scales, and coefficients of consistency (Cronbach's α).

**Scales**	**PSQI**	**ISI**	**JSS**	**QLQ-C30-sleep**	**PHQ-9-sleep**
PSQI		0.79	0.68	0.74	0.69
ISI			0.84	0.77	0.74
JSS				0.68	0.67
QLQ-C30-sleep					0.64
QLQ-C30 global health/QoL	−0.41	−0.45	−0.38	−0.34	−0.36
QLQ-C30 sum score	−0.64	−0.66	−0.54	−0.64	−0.53
PHQ-9 (depression)	0.64	0.70	0.57	0.55	0.65
GAD-7 (anxiety)	0.49	0.54	0.43	0.40	0.40
FoP-Q-SF (fear of progression)	0.45	0.51	0.40	0.41	0.39
CARQ-4 (fear of recurrence)	0.44	0.50	0.39	0.43	0.42
Whiteley index (health anxiety)	0.53	0.58	0.45	0.47	0.46
Cronbach's α	0.79	0.90	0.80	–	–

Within the multi-item questionnaires PSQI, ISI and JSS, the mean correlation was 0.77, while that between the multi-item questionnaires and the single-item scales was somewhat weaker (0.71). Among the additional variables, depression (PHQ-9) and QoL (EORTC QLQ-C30 sum score) were most strongly associated with sleep problems, while the associations with anxiety, fear of progression, health anxiety, and general health/QoL were somewhat weaker for all sleep scales.

The ISI and the PSQI were more strongly correlated with the other scales than the JSS and the two one-items sleep scales. The reliability coefficients (Cronbach's α) of the three multi-item sleep scales were as follows: PSQI: α = 0.79, ISI: α = 0.90, and JSS: α = 0.80.

### Factor analysis of the sleep items

The highest eigenvalues were 10.29, 1.69, 1.40, and 0.92. This means that three eigenvalues exceeded the threshold of 1 according to the Kaiser-Guttman criterion. Consequently, we calculated an analysis with three factors which explained a total of 66% variance. The Kaiser-Meyer-Olkin criterion resulted in a score of 0.940. Table 4 shows the loadings of the items after varimax rotation.

**Table 4:** Factor structure of the sleep items.

**Item/ subscale**	**Item/subscale content**	**F1**	**F2**	**F3**
PSQI 1	Subjective sleep quality	**0.59**	0.48	0.28
PSQI 2	Sleep latency	**0.80**	0.14	0.13
PSQI 3	Sleep duration	**0.69**	0.20	0.23
PSQI 4	Sleep efficiency	**0.71**	0.20	0.19
PSQI 5	Sleep disturbances	0.41	0.16	0.42
PSQI 6	Sleep medication use	0.47	0.00	0.07
PSQI 7	Daytime dysfunction	0.00	0.08	**0.82**
ISI 1	Problems falling asleep	**0.82**	0.22	0.28
ISI 2	Problems staying asleep	0.27	**0.84**	0.25
ISI 3	Early awakening	0.24	**0.47**	0.46
ISI 4	Dissatisfaction	0.48	**0.67**	0.29
ISI 5	Functional impairment	0.34	0.28	**0.79**
ISI 6	Noticeability	0.36	0.21	**0.73**
ISI 7	Distress	0.48	0.38	**0.64**
JSS 1	Trouble falling asleep	**0.77**	0.29	0.29
JSS 2	Wake up several times/night	0.04	**0.87**	0.14
JSS 3	Trouble staying asleep	0.18	**0.86**	0.19
JSS 4	Wake up feeling tired	0.32	0.30	**0.71**
QLQ C30-sleep	Have you had trouble sleeping?	**0.63**	0.44	0.33
PHQ-9-sleep	Trouble falling or staying asleep, or sleeping too much	0.48	0.49	0.42

Bold: loading ≥ 0.50.

Factor 1 is mainly determined by items that indicate problems falling asleep. Factor 2 is characterized by items regarding staying asleep (ISI: problems staying asleep; JSS: wake up several times and trouble staying asleep). Factor 3 is defined by items on functional impairment and daytime dysfunction (PSQI: daytime dysfunction; ISI: functional impairment; JSS: Wake up and feeling tired).

Most questions on general sleep quality (PSQI: subjective sleep quality; ISI: dissatisfaction; C30-sleep item and PHQ-9-sleep item) share components of Factor 1 and Factor 2.

Regarding the three multi-item questionnaires, two of them, the ISI and the JSS cover all three scales with their items. The PSQI, however, is focused on Factor 1, and one item (daytime dysfunction) clearly corresponds to Factor 3.

## Discussion

The first aim of this study was to assess the general sleep quality experienced by PCa patients. Though sleep problems are also common in the general population, the amount of sleep problems was much higher in the PCa sample. According to the relevant cutoff scores, the percentages of poor sleep were 54 % (PSQI), 37 % (JSS) and 57 % (ISI). These figures also illustrate that assessments with different questionnaires can arrive at different proportions of poor sleepers, since the cutoff scores of the questionnaires are located at different levels. To express the burden of sleep problems in clinical samples such as PCa patients, it might be preferable to use effect sizes that refer to the comparison between the clinical group and a corresponding sample of the general population. These effect sizes can only be calculated if normative scores do exist, which was the case for the PSQI and the JSS. The effect sizes (*d* = 0.69 for the PSQI and *d* = 0.97 for the JSS) show that PCa patients showed markedly poorer sleep than their male counterparts in the general population.

However, a meta-analysis comparing different cancer types reported that PCa patients were less strongly affected by sleep problems (44.8 %) than patients with other cancer types (between 50.6 % and 64.4 %) (Al Maqbali et al., 2022). Here one has to take into account that men generally report better sleep quality than women, as can be shown in several normative studies (Tibubos et al., 2020; Hinz et al., 2017a). When assessing the symptom burden perceived by patients, it is important to clarify whether the comparison also considers age and gender differences or not.

Effect sizes can also be used to illustrate the level of burden in the PCa group due to sleep problems in comparison with other symptoms or variables. The effect sizes for the comparison between the PCa patients and the general population were *d* = 0.19 for self-assessed global health, *d* = 0.36 for depression, and 0.35 for anxiety. This also means that, in comparison with other variables of physical and mental health, detriments in sleep quality feature relatively strongly in the PCa group. The relatively low effect size of the difference in self-reported global health (*d* = 0.19) is surprising at first glance. One reason for this may be that the patients were relatively old on average, with the majority being pensioners. In terms of self-reported global QoL, the mean values in the general population decrease with age (Hinz et al., 2014). In cancer patients, the opposite trend can be seen, with lowest general QoL values among younger patients (Hinz et al., 2017b). This means that the difference between the general population and cancer patients is smaller in older age groups. This effect is particularly evident in the self-assessment of overall QoL, while it is less pronounced in the assessment of specific components. The relatively low effect size (*d* = 0.19) obtained for the difference between cancer patients and the general population in comparison with the effect sizes of specific components of QoL has also been confirmed in a study that explicitly compared global components with specific components of QoL (Hinz et al., 2017b). While the overall assessment of QoL tends to depend on personality traits, which are only slightly altered by an event such as cancer, such personality traits have little influence on the assessment of specific symptoms as recorded by the EORTC QLQ-C30 symptom scales (Hinz et al., 2017b).

The high levels of sleep problems in PCa patients mean that identifying and addressing sleep disturbances is crucial for improving the QoL and potentially the clinical outcomes for these patients. Interventions aimed at enhancing sleep quality, such as cognitive behavioral therapy for insomnia or mindfulness-based stress reduction, may offer beneficial effects (Dickerson et al., 2014; Grégoire et al., 2018).

A particular feature of this investigation was the use of several established instruments for measuring sleep quality. We used three multi-item instruments and two single item measures that are included in the questionnaires EORTC QLQ-C30 and in the PHQ-9. The mean correlations between the multi-item questionnaires was 0.77, which means that one questionnaire explains about 59 % of the variance of another questionnaire. While the association between the ISS and the JSS was relatively strong (*r* = 0.84), the PSQI showed a higher degree of independence. This can probably be attributed to the fact that the PSQI explicitly comprises several aspects of sleep quality. It should be noted here that the correlations reflect associations and not agreement. It is difficult to compare our results with other results from the literature, because the parallel use of several questionnaires is rare in sleep-related research.

The internal consistency (Cronbach's α) was sufficient for all three multi-item questionnaires. The lower α coefficient of the PSQI in comparison with the other questionnaires is probably due to the clear multidimensional structure of this questionnaire. While the items of the ISI and the JSS are more homogenous, the PSQI explicitly aims to assess multiple aspects of sleep quality, including subjective assessment, temporal components (sleep duration, sleep latency), functional components (daytime sleepiness) and medication use.

When comparing the three multi-item sleep scales with the single-item scales, the correlations of the multi-item scales were higher than those of the single-item scales in all respects: correlations between the sleep assessment instruments and correlations with other variables of mental and physical health. To a certain degree, it is possible to assess sleep problems with only one item, as was shown in an exemplary study with eight samples of cancer patients (Hofmeister et al., 2022). However, the price for the brevity of the scale is lower reliability. A multi-item questionnaire appears to be necessary for the individual recording of sleep problems, while single-item instruments may be sufficient for epidemiological studies.

The correlations between sleep problems and depression (*r* between 0.55 and 0.70) were higher than the correlations with other variables of mental health, a result that was also found in other studies (Hofmeister et al., 2020). These correlations should be interpreted with caution. Depression and sleep problems have a certain symptom overlap, which is also reflected in the fact that the PHQ-9 depression questionnaire includes an item on sleep problems. This sleep item also contributes to the high correlation between PHQ-9-Sleep and the PHQ-9 total score (*r* = 0.65), in comparison with the correlation between QLQ-C30-Sleep and the PHQ-9 total score (*r* = 0.55). Moreover, it is possible that depressive patients tend to experience and to evaluate all aspects of their life, including sleep quality, in a negative way, and that the high association between depression and subjectively perceived sleep problems can be, at least in part, traced back to such a judgment effect, and which may explain that sleep problems correlate more strongly with depression than with anxiety. Here it is important to note that we only used subjective measurements of sleep quality. In recent years, objective measurements based on actigraphy have increasingly been considered (Chen et al., 2018; Grutsch et al., 2011). Generally, the associations between these objective measures and the questionnaire-based subjective measurements are low (Li et al., 2024), and further research is necessary to clarify the relationship between subjective and objective aspects of sleep quality.

The last research question concerned the dimensional structure of the items underlying the sleep-related questionnaires. Because we did not hypothesize the factor structure and the number of factors in advance, we analyzed the structure with a principal component analysis (PCA). The sequence of the eigenvalues and the Kaiser-Meyer-Olkin criterion (0.940) justify a three-dimensional solution. The three factors can be interpreted as follows: falling asleep and related components (Factor 1), staying asleep (Factor 2), and functional impairment including daytime dysfunction (Factor 3). The ISI and the JSS cover all three factors, while the PSQI focuses on Factor 1. A three-factor solution of the seven components of the PSQI, based on a general population sample, yielded three underlying factors, called sleep efficiency, sleep latency, and sleep quality (Jia et al., 2019), which only partly correspond to the dimensions obtained in this study. Such factors might be useful when comparing subjective assessments of components of sleep with corresponding objective components (Barsasella et al., 2021; Chen et al., 2018).

Some limitations of this study should be mentioned. The sample of PCa patients was taken from the participants of a cancer rehabilitation program and may not be representative for all PCa patients. Patients with low levels of disturbances as well as patients with very high levels might be underrepresented. However, a large study with mixed cancer patients showed that there were only small differences in mean QoL scores obtained in the three settings: inpatient, outpatient, and rehabilitation (Hinz et al., 2018). A further limitation is that it was a monocentric study.

Though we used several frequently used instruments for measuring sleep problems, there are also other instruments which could have been considered here. The associations between sleep problems and other variables, such as anxiety and depression, are only correlative in nature; causal relationships cannot be derived. The factor analysis was based on one specific criterion for the number of factors on one rotation method; other criteria might have resulted in other dimensional structures. Since we have not previously hypothesized about the factor structure, we could not perform confirmatory factor analyses.

In summary, this study showed that sleep problems in PCa are of great importance and efforts should be made to identify and address these problems. The comparison of the various questionnaires for recording sleep quality has shown that each instrument has its own special features, so that the results obtained with a single instrument can only be generalized to a limited extent.

## Data Availability

The raw data supporting the conclusions of this article will be made available by the authors, without undue reservation.
